# Elongation Factor Tu and Heat Shock Protein 70 Are Membrane-Associated Proteins from *Mycoplasma ovipneumoniae* Capable of Inducing Strong Immune Response in Mice

**DOI:** 10.1371/journal.pone.0161170

**Published:** 2016-08-18

**Authors:** Fei Jiang, Jinyan He, Nalu Navarro-Alvarez, Jian Xu, Xia Li, Peng Li, Wenxue Wu

**Affiliations:** 1 Laboratory of Rapid Diagnostic Technology for Animal Disease, College of Veterinary Medicine, China Agricultural University, Beijing, 100193, P. R. China; 2 Key Laboratory of Animal Epidemiology and Zoonosis, Ministry of Agriculture, College of Veterinary Medicine, China Agricultural University, Beijing, 100193, P. R. China; 3 Center For Transplantation Sciences, Massachusetts General Hospital, Harvard Medical School, Charlestown, MA, 02129, United States of America; Boston University Medical School, UNITED STATES

## Abstract

Chronic non-progressive pneumonia, a disease that has become a worldwide epidemic has caused considerable loss to sheep industry. *Mycoplasma ovipneumoniae* (*M*. *ovipneumoniae*) is the causative agent of interstitial pneumonia in sheep, goat and bighorn. We here have identified by immunogold and immunoblotting that elongation factor Tu (EF-Tu) and heat shock protein 70 (HSP 70) are membrane-associated proteins on *M*. *ovipneumonaiea*. We have evaluated the humoral and cellular immune responses *in vivo* by immunizing BALB/c mice with both purified recombinant proteins rEF-Tu and rHSP70. The sera of both rEF-Tu and rHSP70 treated BALB/c mice demonstrated increased levels of IgG, IFN-γ, TNF-α, IL-12(p70), IL-4, IL-5 and IL-6. In addition, ELISPOT assay showed significant increase in IFN-γ^+^ secreting lymphocytes in the rHSP70 group when compared to other groups. Collectively our study reveals that rHSP70 induces a significantly better cellular immune response in mice, and may act as a Th1 cytokine-like adjuvant in immune response induction. Finally, growth inhibition test (GIT) of *M*. *ovipneumoniae* strain Y98 showed that sera from rHSP70 or rEF-Tu-immunized mice inhibited *in vitro* growth of *M*. *ovipneumoniae*. Our data strongly suggest that EF-Tu and HSP70 of *M*. *ovipneumoniae* are membrane-associated proteins capable of inducing antibody production, and cytokine secretion. Therefore, these two proteins may be potential candidates for vaccine development against *M*. *ovipneumoniae* infection in sheep.

## Introduction

*Mycoplasma ovipneumoniae* is the causative agent of chronic non-progressive pneumonia in sheep, goat, bighorn and wild small ruminants [1–4]. It has been a worldwide epidemic that has caused huge economic loss to the sheep industry for decades [1, 3, 5]. Progressive wasting, spasmodic cough, diarrhea and anemia are some of the characteristic symptoms of the disease [6]. Previous investigations led to conclusions that *M*. *ovipneumoniae* was primarily an epizootic pathogen, which could cause secondary infection in fatal respiratory diseases [7–9]. On the other hand, multiple strains of *M*. *ovipneumoniae* have been isolated from infected flock in different countries [2, 6, 10, 11]. Gene polymorphism studies have shown differences in immunogenicity and toxicity among various *M*. *ovipneumoniae* strains. These disparities have only made the research harder, and has slowed down the progress of vaccine development [2].

*M*. *ovipneumoniae* is closely related to *M*. *hyopneumoniae* based on partial sequences of the 16s rRNA gene and HSP70 (DnaK) gene [12, 13]. Genomic sequencing of *M*. *ovipneumoniae* strain SC01 and NM2010 has led to a better understanding of the role of virulent genes in mycoplasma’s pathogenesis [14, 15]. The data showed *M*. *ovipneumoniae* has some adhesion-like protein homologues that are closely related to *M*. *hyopneumoniea*, which include a *M*. *hyopneumoniae* P97-like protein. P97 from *M*. *hyopneumoinae* has been reported to be an outer membrane-associated protein and recombinant P97 protein has been shown to elicit immune response in pigs [16–18]. Shahzad et al. suggested that surface-exposed membrane proteins could suppress the activity of *M*. *ovipneumoniae* [19]. Lack of cell wall may further facilitate mycoplasma’s membrane and cytoskeleton proteins to actively participate during infection of host cells. So here we postulate that the membrane-associated proteins of *M*. *ovipneumoniae*, especially the membrane adhesion-like protein, maybe a potential candidate to design a vaccine against *M*. *ovipneumoinae*.

Elongation factor-Tu (EF-Tu) is one of the most abundant and conserved bacterial proteins. It is involved in the protein synthesis and translation process in prokaryotic cells [20]. EF-Tu is a component of the bacterial membrane cytoskeleton involved in adhesion to the host cells in several pathogenic bacteria [21]. In *M*. *pneumonia*, EF-Tu is surface-localized and may mediate the binding of *M*. *pneumoniae* to fibronectin [22]. Recent studies have demonstrated that EF-Tu was also a bacterial virulence factor and could be an immunodominant protein [23–26].

Heat shock protein (HSP70), a molecular chaperone is one of the most conserved proteins [27]. Recent studies demonstrated that HSP70 could induce both innate and acquired immune responses, and also act as Th1 cytokine-like adjuvant in immune responses [28, 29]. In *M*. *pneumonia*, HSP70 is suggested to play an important role in the organization of the terminal organelle, critical for adhesion to host cells [30].

In this study, we demonstrated that EF-Tu (44 kDa) and HSP70 (66.4 kDa) proteins were *M*. *ovipneumoniae* membrane-associated proteins. The immunogenicity of recombinant EF-Tu (rEF-Tu, a 44.8 kDa protein from a *M*. *ovipneumoniae* wild strain Mo-1) and recombinant HSP70 (rHSP70, a 67.1 kDa protein from a *M*. *ovipneumoniae* wild strain Mo-1) were evaluated in BALB/c mice. Moreover, growth inhibition test (GIT) results indicated that growth of *M*. *ovipneumoniae* was inhibited by the specific antisera *in vitro*.

## Materials and Methods

### Ethics statement

This study was approved by the Beijing Association for Science and Technology (approval ID SYXK (Beijing) 2012–0037) and it was in compliance with Beijing Laboratory Animal Welfare and Ethics guidelines as issued by the Beijing Administration Committee of Laboratory Animals. All animal studies were performed in accordance with the China Agricultural University Institutional Animal Care and Use Committee guidelines (ID: SKLAB-B-2010-003) and approved by animal welfare committee of China Agricultural University.

### *Mycoplasma ovipneumoniae* strains and culture conditions

*Mycoplasma ovipneumoniae* wild strain Mo-1 was isolated from Xinjiang province in China and preserved by the lab. *M*. *ovipneumoniae* strain Y98 was purchased from China Institute of Veterinary Drugs Control. Wild-type strains 2–14, 2–28, 2–37, 5–46, 6–9, 6–11, 7–23, 8–1, 8–5, 8–26 were all field isolates from Heilongjiang, Shandong, Henan and Hebei province, China, 2014.

Medium for *M*. *ovipneumoniae* was prepared with Tryptic Soy Broth (30 g/L), Lactose (10 g/L), Yeast extract (100 mL/L), Penicillin (200 U/mL), Porcine serum 20% (w/v) and 0.4% Phenol Red (w/v), and pH adjusted to 7.4–7.6 with 1 mM NaOH. This medium is a modified version of a medium described previously for culturing the *M*. *ovipneumoinae*[31]. The agar medium of *M*. *ovipneumoniae* contained 1.5% (w/v) agar. *M*. *ovipneumoniae* was cultivated at 37°C with 5% CO_2_, harvested at a mid-log phase by centrifugation at 12,000×g for 20 min at 4°C, washed three times with 0.01 M PBS and stored at -80°C.

### Expression and purification of EF-Tu and HSP 70 proteins

For cloning experiments, EF-Tu and HSP70 specific primers were designed by Primer Premier 5.0 as per HSP70 (DnaK, GenBank No. HM047293.1) of *M*. *ovipneumoniae* strain Y98 and EF-Tu (*Tuf*, GenBank No. JQ990999.1) of *M*. *ovipneumoniae* strain GH3-3. Two restriction sites, Nco I and Xho I, were introduced at both ends of target genes in the flanking primers. These two genes of *M*. *ovipneumoniae* wild strain Mo-1 were amplified by PCR and the amplified products were sequenced. Sequence analysis showed both EF-Tu and HSP70 genes carried the UGA codon. UGA is a termination codon in *Escherichia coli* expression system but codes for tryptophan in Mycoplasma. Therefore, an overlap extension PCR was used to mutate the A of UGA into G in the gene sequence to avoid any truncated gene products. All primers used in this study are shown in the Table 1.

**Table 1:** Primers used for amplification of EF-Tu and HSP70 genes by overlap extension PCR.

Primer No.	Primer name	Primer sequence	Description
1	MoEFTu-NcoI-A	5’CATGCCATGGCAGTTGTTAAAACTGG	Forward primer containing a Nco I site for cloning into pET-28a (+)
2	MoEFTu576-B	5’GTTCAAGAACTTTAGCTTC***CCA***TTCAGGTTTTCCTTCAAG	Reverse primer for site-directed mutagenesis, the mutation site is at genome position 576bp
3	MoEFTu576-C	5’CTTGAAGGAAAACCTGAA***TGG***GAAGCTAAAGTTCTTGAAC	Forward primer for site-directed mutagenesis, the mutation site is at genome position 576bp
4	MoEFTu-XhoI-D	5’CCGCTCGAGTTTAATAATTTCAGTTACTG	Reverse primer containing a Xho I site for cloning into pET-28a (+)
5	MoHSP70-NcoI-A	5’CATGCCATGGGCATGAAAGG	Forward primer containing a Nco I site for cloning into pET-28a (+)
6	MoHSP70-630-B	5’GTCAACAATTTGATTATC***CCA***GTCATCACCACCTAAATGG	Reverse primer for site-directed mutagenesis, the mutation site is at genome position 630bp
7	MoHSP70-651-C	5’GATAATCAAATTGTTGAC***TGG***ATGATTAAAAGAATTAAAG	Forward primer for site-directed mutagenesis, the mutation site is at genome position 651bp
8	MoHSP70-XhoI-D	5’CCGCTCGAGATTTTGTTTGATTTCAGCATC	Reverse primer containing a Xho I site for cloning into pET-28a (+)

The full-length PCR products of mutated target genes were digested by the restriction enzymes Nco I and Xho I. The fragments were then cloned into the PET-28a (+) prokaryotic expression vector to construct the pET-28a (+)-EF-Tu and pET-28a (+)-HSP70 plasmids. Rosetta (DE3) chemically competent cell (TaKaRa, Dalian, China) was used to express the recombinant protein. The competent cells carrying pET-28a (+)-EF-Tu or pET-28a (+)-HSP70 plasmids were grown in sterile Luria-Bertani (LB) media containing kanamycin (25 μg/ml). Mid-log phase growth was achieved before 0.5 mM isopropyl β-D-thiogalactoside (IPTG) induction at 37°C for 6 h. Finally, the harvested cells were disrupted by sonication, and recombinant proteins purified by Ni-NTA columns as per manufacturer’s instruction (Qiagen, Germany). Amicon^®^ Ultra-15 Centrifugal Filter Devices (Merck Millipore, USA, MW cutoff 30 kDa or 50 kDa) were used to remove the imidazole and concentrate the purified recombinant proteins. Then the lipopolysaccharide (LPS) in recombinant proteins was removed using EtEraser Endotoxin Removal Kit (Chinese Horseshoe Crab Reagent Manufactory, Xiamen, China). LPS contamination was determined to be less than 1 EU/ml using the Quantitative Chromogenic Tachypleus Amebocyte Lysate (TAL) for endotoxin detection kit (Chinese Horseshoe Crab Reagent Manufactory, Xiamen, China).

### Immunoblotting of recombinant EF-Tu protein (rEF-Tu) and recombinant HSP70 proteins (rHSP70)

Purified rEF-Tu and rHSP70 proteins were subjected to 12% sodium dodecyl sulfate-polyacrylamide gel electrophoresis (SDS-PAGE) and transferred to polyvinylidene difluoride (PVDF) membranes. The membranes were blocked in PBST with 5% nonfat milk for 1h and then incubated overnight at 4°C with specific sheep *M*. *ovipneumoniae* hyper-immune sera. Following washing with PBST three times, membranes were incubated with rabbit anti-goat IgG HRP- conjugated antibody at 37°C for 1 h and exposed to SuperSignal^®^ West Pico Chemiluminescent Substrate reagent for detection (Thermo Scientific, USA).

### Identification of EF-Tu and HSP70 as membrane-associated protein

#### Immunogold

Fresh intact *M*. *ovipneumoniae* strain Y98 cells were harvested at mid-log phase and washed with 0.01 M PBS three times. The cell pellets were fixed in PBS containing 2% (w/v) paraformaldehyde and 2.5% (w/v) glutaraldehyde and processed as previously described [32]. After embedding in Lowicryl, sample sections were cut, mounted onto Formvar-coated nickel grid, blocked with PBS-Glycine buffer (PBS with 50 mM glycine) for 10 min, and washed with PBGT buffer (PBS with 0.1% gelatin, 1.0% BSA, 0.1% Tween 20) six times. Grids were incubated overnight at 4°C with anti-rEF-Tu or anti-rHSP70 sera (The sera were collected from mice immunized with either recombinant protein respectively 42 days after immunization, both 1:1,000 diluted). After washes with PGBT, grids were incubated at 25°C for 60 min with goat anti-mouse IgG (whole molecule)-Gold (5 nm) antibody (1:20 diluted, Sigma, USA). Samples were counterstained with aqueous uranyl acetate and examined by transmission electron microscope (TEM). TEM measurements were made on an H-7500 (Hitachi, Japan) at 80 kV. Sera from non-immunized mice were used as negative control.

#### Immunoboltting

Membrane proteins of 12 strains of *M*. *ovipneumoniae*, including strain Y98 and Mo-1, were extracted using 1.5%(v/v) Triton X-114. Briefly, 50 mL fresh intact *M*. *ovipneumoniae* cells were harvested at mid-log phase and washed with 0.01 M PBS three times. Then, the *M*. *ovipneumoniae* pellets were suspended in 2 mL 0.01 M PBS containing 1.5% (v/v) Triton X-114, incubated at 25°C for 10 min and centrifuged at 12,000×g for 30 min at 4°C. 2.5 mL cold ethanol was added to the supernatant, mixed well and precipitated overnight at -20°C, and then centrifuged at 12,000×g for 30 min at 4°C. The pellets were suspended in 5.0 mL 0.01 M PBS and centrifuged at 12,000×g for 30 min at 4°C. Finally, the pellets were suspended in 0.5 mL 0.01 M PBS. Immunoblotting was performed in the membrane proteins extracts of 12 strains of *M*. *ovipneumoniae* in order to identify whether EF-Tu and HSP70 were membrane-associated proteins. Mouse anti-rEF-Tu sera or anti-rHSP70 sera collected at 42 DAI (day after immunization), were used as primary antibodies at 1:2,000 dilution. Purified rEF-Tu and rHSP70 proteins were used as the positive controls in Western bolting.

### Grouping and immunization of experimental animals

Seven-eight weeks old SPF female BALB/c mice were randomly divided into four groups. The first three groups were immunized with 1) recombinant protein rEF-Tu, 2) recombinant protein rHSP70 or 3) Mo extracts (the supernatant of Mo-1 whole cell ultrasonic lysates, LPS were less than 1 EU/ml) respectively. The fourth group served as a non-immunized control. Each mouse in each group was inoculated three times subcutaneously with 50 μg of antigen in 100 μL of either Freund’s complete adjuvant (CFA) or Freund’s incomplete adjuvant (IFA) at two week intervals. Antigens mixed and emulsified with the same volume of CFA were used for the first immunizations and antigens with IFA for the next two immunizations. Simultaneously, 100 μL of sterile PBS (pH 7.4–7.6) mixed with respective adjuvant as antigens were injected into each mouse of non-immunized control group.

### Detection of antibody level and antibody subtypes

Mice sera were collected at 3, 7, 14, 21, 28, 35, 42, 49, 56, 63 and 70 DAI. Indirect ELISA was performed to detect IgG titers and subtypes in the immunized groups. Antibody titers were performed in all time point sera, whereas detection of antibody subtypes was performed only in sera at 42 DAI. ELISA plates were coated with purified rEF-Tu proteins or rHSP70 proteins or Mo extracts of *M*. *ovipneumoniae* wild strain Mo-1 at a concentration of 100 ng per well. For antibody titers, a 2-fold serial dilution of sera starting at 1:50 was performed. Goat-anti-mouse IgG (H+L) HRP diluted at 1:5,000 was used as secondary antibody. On the other hand, sera harvested at 42DAI were diluted at 1:1,000, HRP-goat-anti-mouse IgG1 or IgG2a (diluted at 1:500) were used for detection of antibody subtypes. All indirect ELISA was developed using TMB/ H_2_O_2_. Color development was stopped by adding 2M H_2_SO_4_ and plates were read at 450 nm.

### Detection of cytokine and lymphocyte secreting IFN-γ

Six cytokines, IFN-γ, TNF-α, IL-12(p70), IL-4, IL-5 and IL-6, in sera of immunized mice collected at 3, 7, 14 and 35 DAI were detected using Bio-Plex Pro^™^ mouse cytokine assay (Bio-Rad, USA). The levels of IFN-γ^+^ secreting lymphocytes were detected by Mouse IFN gamma ELISPOT Kit (affymetrix eBioscience, USA). Spleen lymphocytes of immunized mice were harvested at 35 DAI and plated (100,000 cells per well) on the ELISPOT plate. The lymphocytes were stimulated with rHSP70 (5 μg/well), rEF-Tu (5 μg/well), Mo extracts (5 μg/well), PHA (0.5 μg/well) or PBS, respectively and incubated at 37°C with 5% CO_2_ humidified incubator for 48 hours. Finally, spots were counted using the automated ELISPOT plate reader.

### *In vitro M*. *ovipneumoniae* Growth inhibition test

In order to test the immunogenicity of the two proteins *in vitro*, GIT were performed in this study.

#### GIT on agar medium

Mycoplasma agar medium was melted and poured into culture dishes with a diameter of 60 mm. *M*. *ovipneumoniae* suspension of 100 μL (10^6^ CCU/mL) was inoculated and distributed uniformly onto each dish after its solidification. After incubation at 37°C for 1 h, 2.00 mm diameter paper discs saturated with 10 μL of undiluted antisera (rEF-Tu antisera, rHSP70 antisera, Mo Extracts antisera or non-immune control (PBS) sera collected at 70 DAI) were placed on the agar. Dishes were incubated at 37°C with 5% CO_2_ for 7 days and then the diameter of inhibition zones were measured.

#### GIT in liquid medium

A modified assay was performed as described previously[33]. Briefly, 100 μL (10^6^ CCU/mL) *M*. *ovipneumoinae* strain Y98 were poured into 96-well microplates. Then, 100 μL of serial dilutions (1:20–1:2,560) of each group sera (collected at 70 DAI) were then added into wells in triplicate. Mycoplasma that cultured in the absence of serum severed as a positive control. As no antiserum control, only the culture medium was in the wells. Absorbance was recorded at 560 nm after incubation at 37°C with 5% CO_2_ for 7 days. The percentage of growth inhibition was determined as follows: [absorbance of sample − absorbance of positive control]/[absorbance of no antiserum control − absorbance positive control]×100%.

### Statistical analysis

Levels of antibodies and concentrations of cytokines between different groups were compared using One-way ANOVA (SPSS 17.0 and GraphPad Prism 6). *P* values below 0.05 were considered statistically significant.

## Results

### Expression and identification of recombinant EF-Tu and HSP70

**Fig 1:**
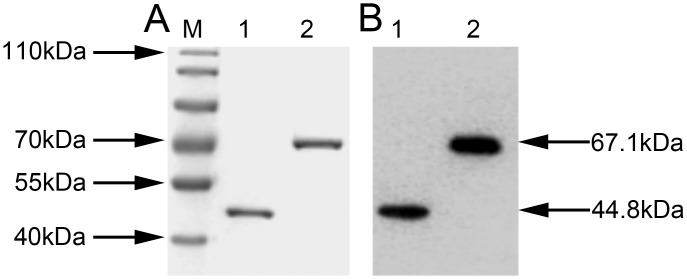
Expression and identification of recombinant EF-Tu/HSP70. (A) Purity analysis of the recombinant His-tagged rET-Tu and rHSP70 proteins by 12% SDS-PAGE. Lane M, Thermo Scientific^™^ PageRuler^™^ Prestained Protein Ladder (10 to 180kDa); Lane 1, purified rEF-Tu protein; Lane 2, purified rHSP70 protein. (B) The immunoblot of rET-Tu and rHSP70 with sheep *M*. *ovipneumoniae* hyper-immune sera. Lane 1, purified rEF-Tu protein; Lane 2, purified rHSP70 protein.

EF-Tu (*Tuf*) gene (1209bp) and HSP70 (DnaK) gene (1818bp) of *M*. *ovipneumoniae* wild strain Mo-1 were obtained by site-mutation (UGA to UGG) and overlap extension PCR assay (S1 Fig). The two expressed recombinant proteins were purified with Ni-NTA. The SDS-PAGE results showed that rHSP70 protein was about 67.1 kDa and rEF-Tu protein was about 44.8 kDa (Fig 1A). The two purified recombinant proteins could be recognized by sheep hyper-immune sera against *M*. *ovipneumoniae* strain Y98 in immunoblotting (Fig 1B).

### Confirmation of HSP70 and EF-Tu as membrane-associated proteins

**Fig 2:**
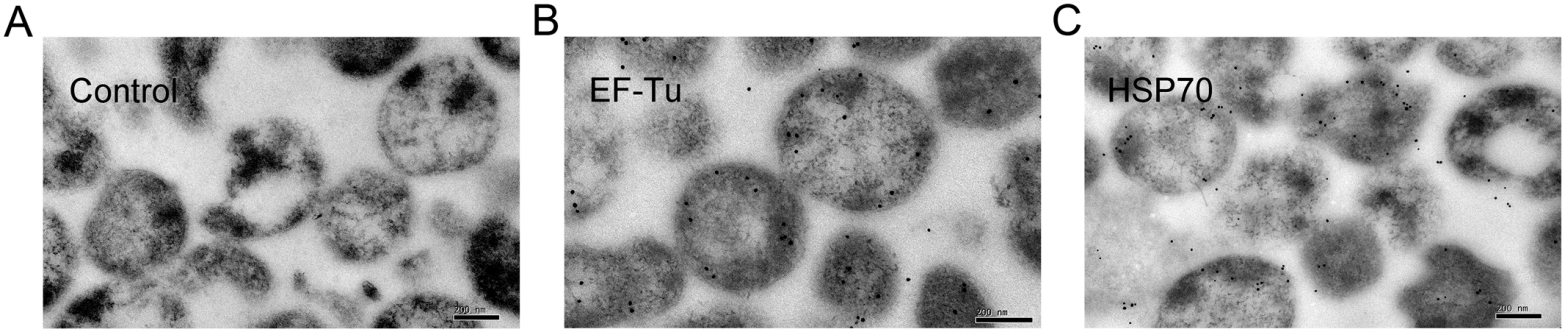
Immunogold labeling of EF-Tu and HSP70 protein on *M*. *ovipneumoniae* Y98 strain. *M*. *ovipneumoniae* whole cell normal control (A), *M*. *ovipneumoniae* incubated with anti-rEF-Tu sera (B) and *M*. *ovipneumoniae* incubated with anti-rHSP70 sera (C) are displayed. *M*. *ovipneumoniae* were incubated with mouse anti-rEF-Tu/anti-rHSP70 sera generated against truncations followed by anti-mouse IgG gold complex (5 nm). Bar, 200 nm.

**Fig 3:**
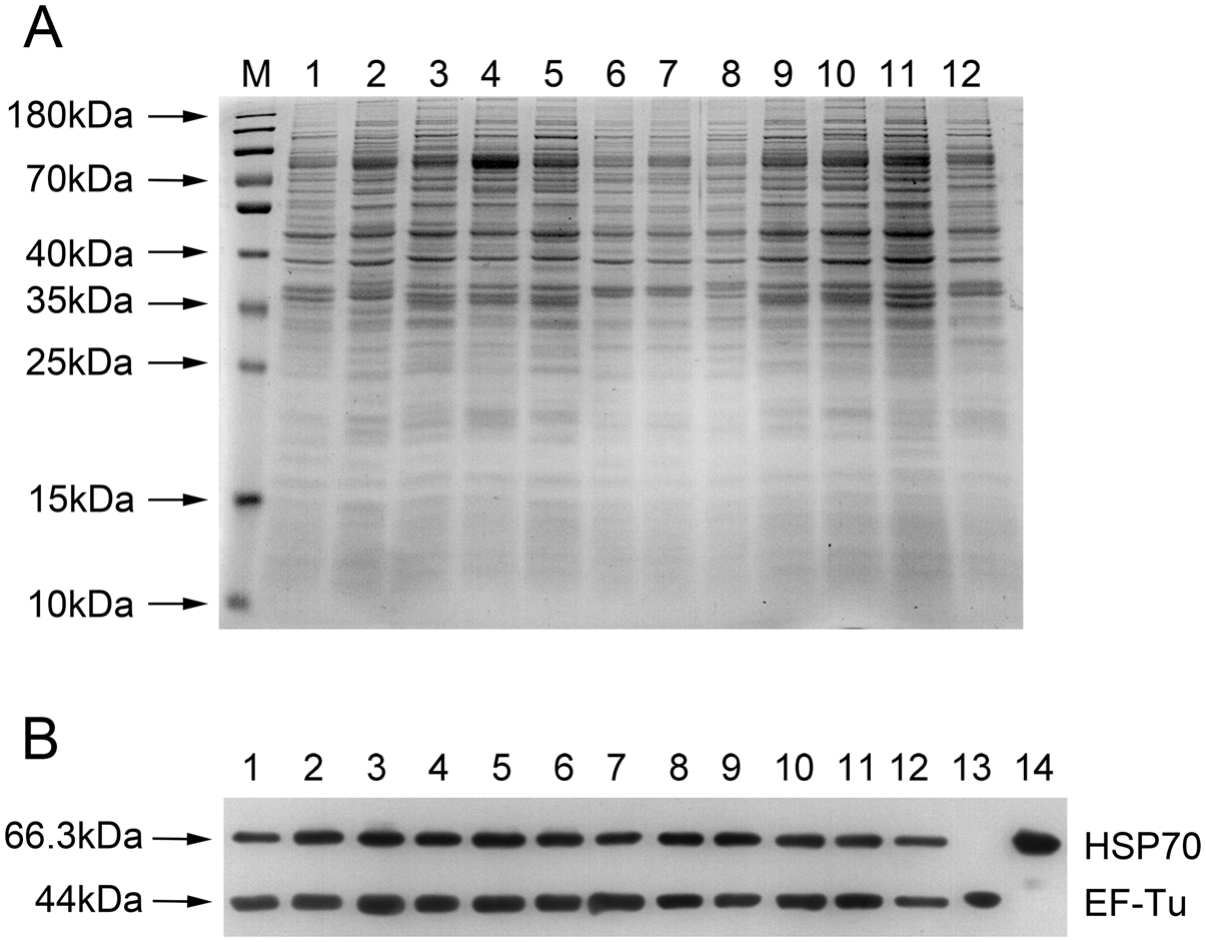
Comparison of the whole cell proteins and membrane proteins of *M*. *ovipneumoniae* strain Y98, strain Mo-1, and wild-type strains by SDS-PAGE and immunoblots. Extracted membrane proteins of 12 different *M*. *ovipneumoniae* strains were separated by 12% SDS-PAGE (A) and subjected to Western blotting analysis with the mouse anti-rEF-Tu/anti-rHSP70 sera (B). Lane M, Thermo Scientific^™^ PageRuler^™^ Prestained Protein Ladder (10 to 180kDa); Lane 1, Mo-1; Lane 2, Y98; Lane 3–12, *M*. *ovipneumoniae* wild strains 2–14, 2–28, 2–37, 5–46, 6–9, 6–11, 7–23, 8–1, 8–5, 8–26; Lane 13, purified rEF-Tu protein control, lane 14: purified rHSP70 protein control.

Immunogold staining and imaging by transmission electron microscope (TEM) were performed to locate EF-Tu and HSP70 to verify whether the two proteins were membrane-associated proteins. Based on the observations of EF-Tu and HSP70 in TEM micrographs, most *M*. *ovipneumoniae* presented circular morphology with immunogold staining mainly localized around *M*. *ovipneumoniae* cells after incubation with anti-rHSP70 or anti-rEF-Tu antibody. The observations thus indicated that the two proteins were indeed membrane-associated proteins (Fig 2). In order to locate EF-Tu and HSP70 on *M*. *ovipneumoniae* and to verify whether they were membrane associated proteins of *M*. *ovipneumoniae*, membrane proteins of 12 strains of *M*. *ovipneumoniae*, including Y98, Mo-1 and 10 wild-type strains were extracted using 1.5% (v/v) Triton X-114. Immunoblotting was performed with membrane proteins of 12 strains of *M*. *ovipneumoniae*, and the results confirmed presence of EF-Tu and HSP70 in extracted membrane proteins (Fig 3).

### Analysis of antibody level and subtypes in sera of immunized mice

**Fig 4:**
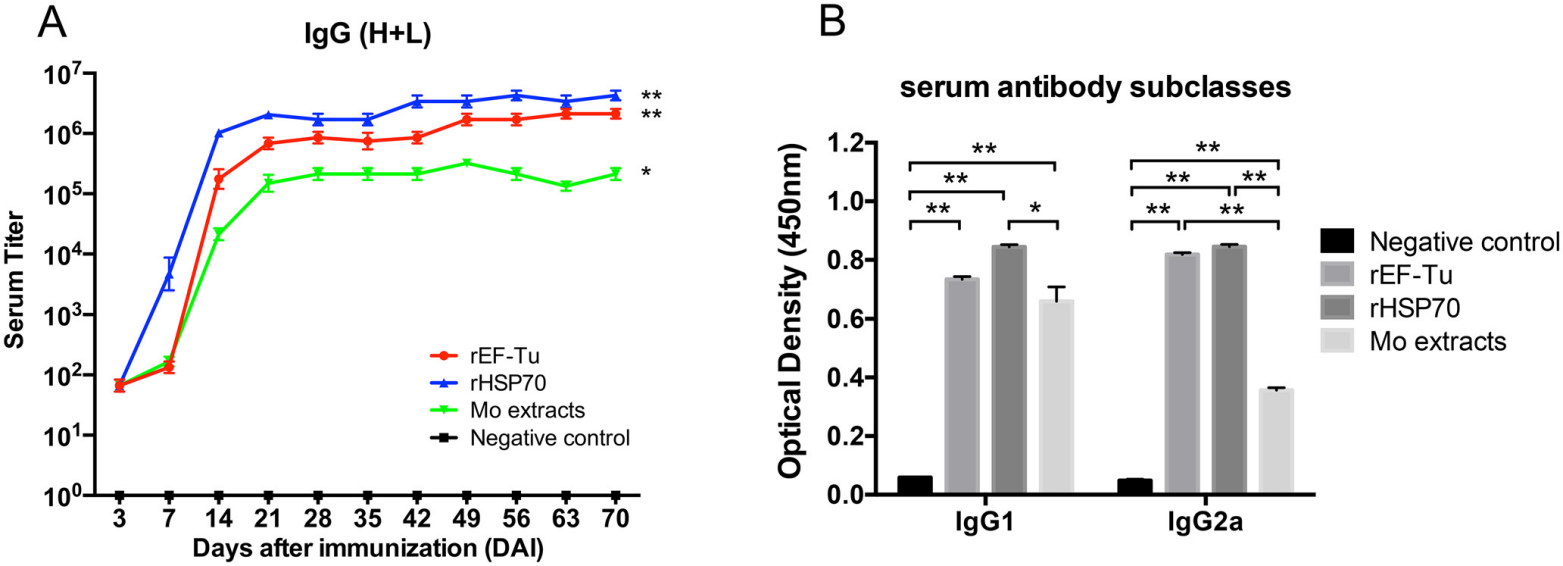
The immunized mouse serum IgG (H+L) titer and IgG subtypes were detected using the indirect ELISA assay. (A) IgG titers of BALB/c mice immunized with rEF-Tu, rHSP70, Mo extracts and PBS sera of each groups were collected on days 3, 7, 14, 21, 28, 35, 42, 49, 56, 63 and 70 DAI. (B) Determination of IgG subtypes in sera of the immunized mice. The sera of each group were collected at 35 DAI. ELISA plates were coated with purified rEF-Tu proteins or rHSP70 proteins or Mo extracts of *M*. *ovipneumoniae* wild strain Mo-1 at a concentration of 100 ng per well. Anti-mouse IgG (H+L) or IgG1 or IgG2a HRP-conjugated antibodies were used as secondary antibodies. Asterisks indicates the results of the One-Way ANOVA using the Tukey test, compared with the PBS negative control group, with *P* < 0.01(**) or *P*<0.05(*), n = 6.

IgG titer in sera of BALB/c mice were detected by indirect ELISA using rEF-Tu, rHSP70 or Mo extracts as the coating antigens (Fig 4A). Results showed that mice immunized with rEF-Tu, rHSP70 or Mo extracts proteins elicited strong humoral responses with the rHSP70-immunized mice showing higher levels of antibody than rEF-Tu or Mo extract groups at 7 DAI. However, the differences among the three groups were not significant (p>0.05). At 14 DAI, the IgG levels in sera of three immunized groups were significantly increased especially from the rHSP70 group. The rHSP70 and rEF-Tu groups showed higher antibody titers compared to Mo extracts group, and the significant differences (p<0.01) lasted till 70 DAI. The difference between rHSP70 and rEF-Tu, however, were not significant (p>0.05). With the exception of the PBS negative control group, all the immunized groups could generate IgG1 and IgG2a simultaneously. There was no significant difference in the amount of IgG1 and IgG2a elicited by either rHSP70 or rEF-Tu, however they elicited a significantly higher IgG2a production when compared with Mo extracts group (Fig 4B).

### Detection of cytokines in sera of mice at 3, 7, 14 and 35 DAI

Six cytokines including Th1 type cytokines (IFN-γ, TNF-α, IL-12p70) and Th2 type cytokines (IL-4, IL-5, IL-6) in sera of mice at 3, 7, 14 and 35 DAI were analyzed.

**Fig 5:**
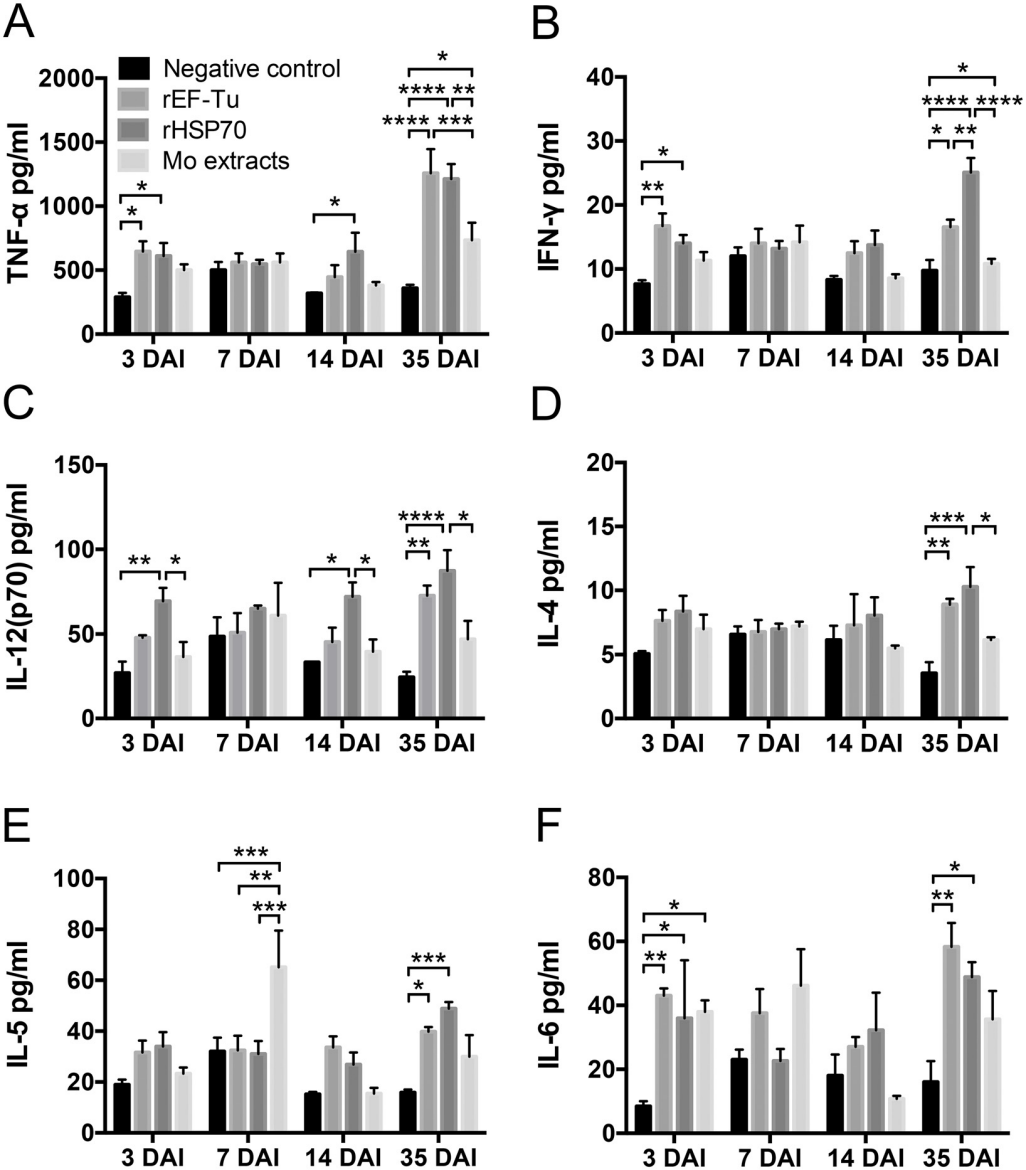
The concentration of essential Th1/Th2 cytokines in the serum of immunized mice. Serum from BALB/c mice immunized with rEF-Tu, rHSP70, Mo extracts or PBS were collected at 3, 7, 14 and 35 DAI, respectively. The data show the TNF-α, IFN-γ, respectively. The data show the expression levels (pg/ml) in the immunized mouse serum. All data represent the mean ± SEM (n = 6). Treatments that differed from the each groups according to the One-Way ANOVA using Tukey test. *P*<0.05 (*), *P*<0.01 (**) or *P*<0.001 (***) depicts the significance value.

While TNF-α, IL-12(p70) and IL-6 were chosen for their early immune response participation, IFN-γ, IL-4 and IL-5 levels were analyzed to assess the progression of the immune response induced by the recombinant proteins 14 days after the first immunization (Fig 5).

At 3 DAI, all three immunized groups showed higher levels of all six cytokines compared to the negative control group. The concentrations of IFN-γ and TNF-α in rHSP70 and rEF-Tu groups were significantly higher than the negative control group (p<0.05 or p<0.01). rHSP70 protein immunized group also showed significantly higher levels of IL-12(p70) than the negative control group. The level of all six cytokines in the Mo extracts group reached their peak concentration at 7 DAI, while the cytokines in rHsp70 and rEF-Tu groups were either slightly decreased or showed no change when compared with 3 DAI. At 14 DAI, all cytokines of rHsp70 group were higher than the other groups with the exception of IL-5.

Seven days after third immunization (35 DAI), all Th1 and Th2 type cytokine concentrations of rHSP70 and rEF-Tu groups were increased and at the highest level compared to 3 DAI, 7 DAI and 14 DAI. rHsp70 induced the higher expression levels of cytokines among four groups (Fig 5). There were significant differences (p<0.05, p<0.01 or p<0.001) between rHsp70 and Mo extracts in all cytokines except IL-6 (Fig 5A–5F). The rHsp70 also induced a higher expression level of IFN-γ compared to rEF-Tu group at 35 DAI (Fig 5B), while the concentration of TNF-α in rEF-Tu group was significantly higher with Mo extracts (p<0.05) (Fig 5A). Besides, although the cytokine levels of Mo extracts group were higher than the negative control, only TNF-α value showed significant difference compared to the negative control (p<0.05) (Fig 5A).

### Detection of lymphocytes secreting IFN-γ by ELISPOT

**Fig 6:**
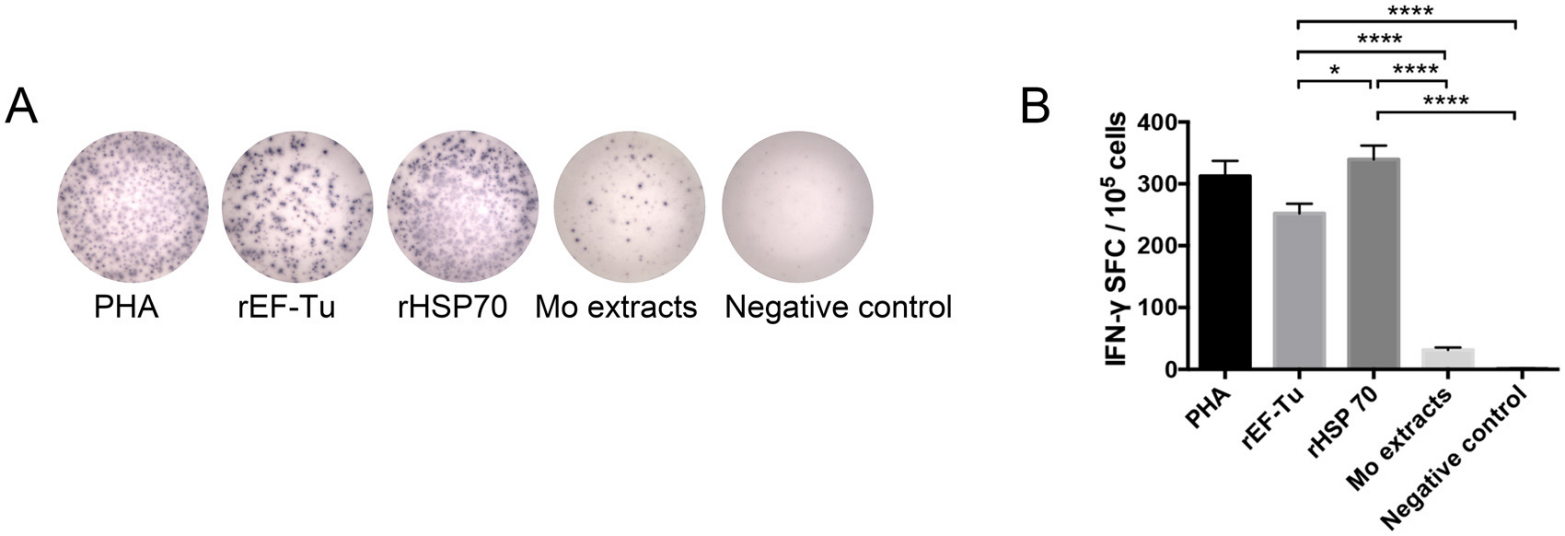
Result of the IFN-γ^+^ secreting spleen lymphocytes in the immunized mice by ELISPOT. Spleen lymphocytes of different immune groups were collected and separated at 35 DAI. Lymphocytes were stimulated with PHA, rEF-Tu, rHSP70, Mo extracts or PBS for 48h. (A) Representative images of the IFN-γ^+^ secreting spleen lymphocytes in different groups are shown. (B) Number of INF-γ^+^ spot-forming cells (SFC) was enumerated by ELISPOT, Comparison of spots between different groups. All data represent the mean ± SEM (n = 6). The *P* value of each pair of comparison was calculated using One-Way ANOVA. *P*<0.05 (*), *P*<0.01 (**), *P*<0.001 (***) or *P*<0.0001 (****) depicts the significance value.

The cellular immune response was assessed by the amount of IFN-γ^+^ secreting lymphocytes using ELISPOT assay. Mice spleen lymphocytes were isolated at 35 DAI and stimulated by rHSP70 or rEF-Tu or Mo extracts for 48h. Each spot represented one IFN-γ^+^ secreting lymphocyte (Fig 6A) and the mean spot of PHA positive control group was 313 in every 100,000 lymphocytes. Fig 6A and the calculated results (Fig 6B) demonstrated that rHSP70 (339/100,000 lymphocytes) and EF-Tu (252/100,000 lymphocytes) stimulation significantly induced IFN-γ secretion of lymphocytes compared to Mo extracts (31/100,000 lymphocytes) or PBS negative control (1/100,000 lymphocytes). Furthermore, the amount of IFN-γ^+^ secreting lymphocytes in rHSP70 group was significantly different from rEF-Tu group, which were in concordance with the results of cytokines test (Figs 5B and 6B).

### Growth inhibition test

**Fig 7:**
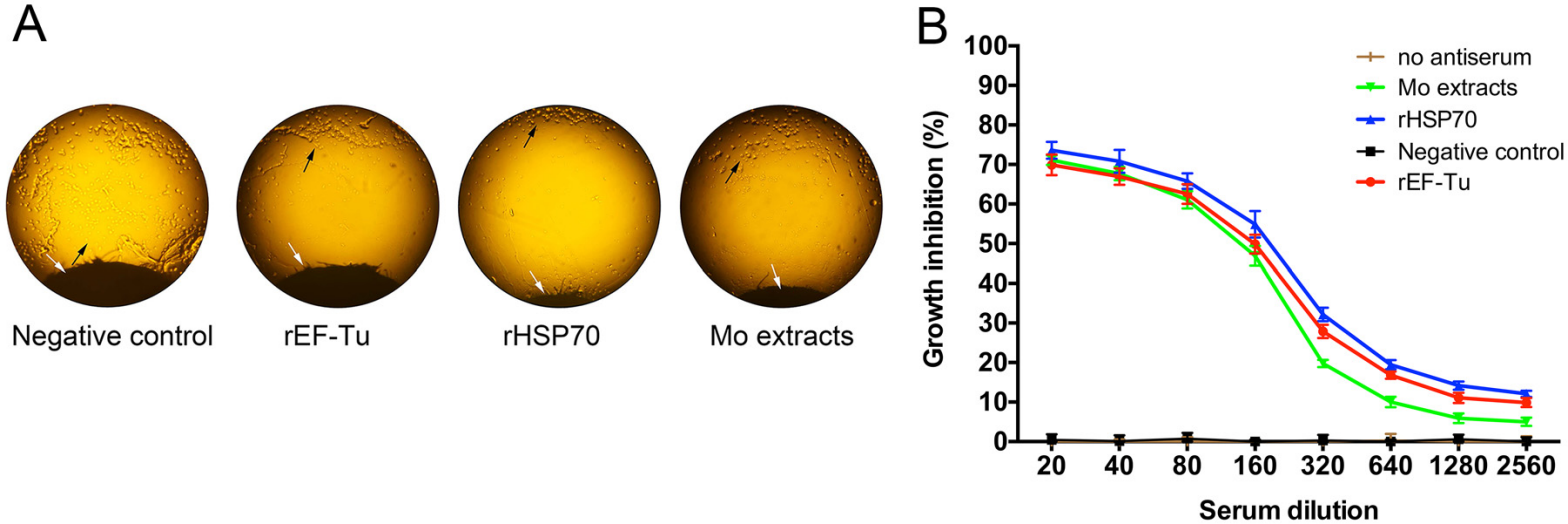
*In vitro* growth inhibition test of *M*. *ovipneumoinae*. GIT test were performed by using mouse rHSP70 antisera, rEF-Tu antisera, Mo extracts antisera or PBS negative control sera (collected at 70 DAI). (A) GIT (on solid medium) photomicrograph of the edge of a paper disc (on the bottom) previously impregnated with specific antiserum. The 2.00 mm diameter paper discs were saturated with 10μl antisera. Adjacent to the disc were the zone of inhibited growth. Black arrow indicates the *M*. *ovipneumoniae* colonies. White arrow indicates the edge of paper disc. (B) The percentage of growth inhibition of *M*. *ovipneumoniae* tested in liquid medium. Mycoplasma cells (10^5^ CCU/well) were cultured in the presence of serial dilutions of antisera in 96 wells plates. The absorbance of growth medium was measured at 560nm. The percentage of growth inhibition was determined as follows: [absorbance of sample − absorbance of positive control]/[absorbance of no antiserum control − absorbance positive control]×100%. All data represent the mean ± SEM, n = 3.

Mycoplasma growth inhibition test was performed to verify immunogenicity of rEF-Tu and rHSP70 with sera from four groups of immunized mice. When compared to the non-immune control (PBS) group, inhibition zones on agar medium appeared in all the three other groups (Fig 7A). The average diameter of inhibition zone in rEF-Tu, rHSP70 and Mo extracts groups was 5.17±0.41 mm,7.00±0.32 mm and 5.5±0.32mm respectively. As shown in Fig 7B, sera from rEF-Tu, rHSP70 and Mo extracts groups immunized groups also inhibited the growth of *M*. *ovipneumoniae* in liquid medium. As expected, the anti-rHSP70 sera showed a higher growth inhibitory activity than two other immunized groups. This result also provides further evidence that the antisera of two recombinant proteins could inhibit the growth of *M*. *ovipneumoniae in vitro*.

## Discussion

The epidemic of *M*. *ovipneumoniae* around the world has caused considerable economic loss to sheep industry. Despite extensive research on *M*. *ovipneumoniae* [2], a potential vaccine is yet to be discovered due to insufficient database on *M*. *ovipneumoniae* pathogenesis. Thus, it is necessary to characterize novel protective antigens for developing *M*. *ovipneumoinae* vaccines. In this study, we confirmed that both EF-Tu and HSP70 were membrane-associated proteins of *M*. *ovipneumoniae* and evaluated the immunogenicity of the two proteins.

Mycoplasma is the smallest and simplest self-replicating organism that contains the minimal complement of life-enabling genes and lacks cell wall which may further facilitate its membrane and cytoskeleton proteins to actively participate during infection of host cells [34]. As an intracellular pathogen, adhesion mediated by membrane-associated proteins is believed to be a significant step in mycoplasma pathogenesis [35, 36]. Therefore, it may be possible to reduce infection of *M*. *ovipneumoniae* by immunization with membrane-associated proteins.

It has been recently reported that EF-Tu was a membrane-associated protein of *M*. *pneumonia* and mediated the fibronectin binding of *M*. *pneumonia* during pathogenesis [22, 23]. HSP70 protein also plays an important role in the organization of the terminal organelle and is critical for adhesion to host cells in *M*. *pneumonia* [30], however, the location of HSP70 on mycoplasma is not clear. Here, we characterized EF-Tu and HSP70 were membrane-associated proteins by immunogold staining and immunoblotting. In addition to the rounded morphology of *M*. *ovipneumoniae* TEM data also indicated that these membrane-associated proteins were not exclusive to the surface alone, but also were intracellular as evidenced by cytoplasmic staining of the proteins. Using extracted membrane proteins of 12 strains of *M*. *ovipneumoniae*, we demonstrated the presence of EF-Tu and HSP70 by Western blotting, which further confirmed both EF-Tu and HSP70 are membrane-associated proteins of *M*. *ovipneumoniae* (Fig 3).

In previous studies, the membrane-associated proteins like P97, P42 and MnuA of *M*. *hyopneumoniae*, P30 of *M*. *pneumoinae*, MGC3 of *M*. *gallisepticum* were determined as the potential antigen candidates for vaccine development [18, 37–40]. EF-Tu of *M*. *hyopneumoniae* and *M*. *bovis* were reported to be potential antigens [24, 41]. Moreover, HSP70 has been reported as an immunogenic biomolecule of mycoplasma that can induce and enhance humoral and cellular immunity [42]. Chen et al. also demonstrated that heat shock protein P42 of *M*. *hyopneumoniae* can be recognized by multiple B-cell and T-cell clones [37]. Taken all together, these studies lend the support to our observations of immunogenic EF-Tu and HSP70 in *M*. *ovipneumoinae*. Here, we demonstrated that EF-Tu and HSP70 of *M*. *ovipneumoniae* induce both humoral and cellular immune responses in mice.

The analysis of the antibody response revealed that Mo extracts, recombinant proteins rEF-Tu and rHSP70 were able to induce specific IgG antibodies, suggesting that these recombinant proteins could elicited a strong humoral immune response in mice. It is known that Th1 cells induce B cells to proliferate and produce antibodies such as IgG1 while Th2 cells induce B cells to produce IgG2a in mice [43]. We determined that both rEF-Tu and rHSP70 had the ability to produce more IgG1 and IgG2a antibodies than Mo extracts group (Fig 4B). Furthermore, the results revealed that both IgG1 and IgG2a in rHSP70 and rEF-Tu groups were induced at similar levels, which indicates that the recombinant proteins may induce a mixed Th1 and Th2 response in the mice.

Additionally, the results of cytokines production test showed that all proteins could active an innate immune response in mice (Fig 5). As an important inflammatory cytokine, TNF-α is the earliest inflammatory cytokine to activate neutrophils and lymphocytes, which can increase the permeability of vascular endothelial cells, adjust tissue metabolism and promote synthesis and release of cytokines[44, 45]. We here observed that rEF-Tu and rHSP70 elicited a significant early increase (3 DAI) in TNF-α levels which doubled at 35 DAI (Fig 5A). This increased TNF-α levels were also observed with Mo extracts when compared with non-immunized mice, but was not as robust as that produced by rEF-Tu or rHSP70. The same early and late cytokine production response was observed for both proteins when measuring IL-12(p70) and IL-6 (Fig 5C and 5F). Based on our results, we suggest that the recombinant proteins could induce a stronger innate immune response than Mo extracts.

In general, recombinant protein vaccines are known to preferentially induce Th2 responses with weak or no Th1 responses. However, it has been reported that some recombinant protein of *M*. *hyopneumoniae* can induce Th1 response in mice[38, 46, 47]. The analysis of the cellular immune responses revealed that rEF-Tu and rHSP70 induced high levels of IFN-γ than Mo extracts group from 3 DAI to 35 DAI (Fig 5B). The ELISPOT assay also demonstrated the ability of both recombinant proteins to induce immune response by secretion of IFN-γ at 35 DAI (Fig 6). As expected, the ELISPOT results corresponded to the cytokine production test. Additionally, since the concentration of IL-4 and IL-5 induced by rEF-Tu and rHSP70 were significantly higher than non-immunized group (Fig 5D and 5E), we suggest that the two recombinant proteins could elicit Th1- and Th2-based immune responses. Furthermore, rHSP70 could re-activated lymphocytes quickly and induce specific cellular immune response according to the higher level of IFN-γ in HSP70 group.

In previous studies, HSP70 has been reported to induce antigen-specific immune responses thereby transducing various signals into dendritic cells (DCs) to enhance immune responses[48]. The C-terminal peptide-binding domain of HSP70 might act as a microbial adjuvant that upregulates the expression of cytokines such as IL-12 and TNF-α. IL-12 (p70) known to be a very potent cytokine for Th1 polarization, and it was the most induced from 3 DAI to 35 DAI in rHSP70 group in our study. Thus we suggest that rHSP70 might also act as a Th1 cytokine-like adjuvant for immune response induction in *M*. *ovipneumoniae*.

Since there is still no applicable laboratory animal model for experimental infection with *M*. *ovipnemoniae* with the exception of the sheep model, which is a tedious model to work with, we here performed *M*. *ovipneumoniae* GIT to verify the immunogenicity of the two protein specific antibodies (Fig 7). Herein, the experiments *in vitro* proved that antisera of mice immunized with rEF-Tu or rHSP70 could inhibit growth of *M*. *ovipneumonia* strain Y98 and the rHSP70 antiserum was more potent at inhibiting the growth of *M*. *ovipneumoniae* than the other ones. These results may provide us a critical marker to evaluate potential vaccine antigens of *M*. *ovipneumoinae*.

In summary, the results of this study showed that both HSP70 and EF-Tu were membrane-associated proteins of *M*. *ovipneumoniae*, and the recombinant proteins were able to induce Th1 and Th2 based immune responses elicited to produce high levels of IgG1 and IgG2a with increased INF-γ expression. Additionally, compared with the three immunized groups, rHSP70 protein could induce the highest level of IFN-γ expression and anti-HSP70 protein antibody showed a better ability inhibiting *M*. *ovipneumoniae* growth. The results demonstrated that both rHSP70 and rEF-Tu antigens might be good candidates for the development of *M*. *ovipneumoniae* subunit vaccine. However, it should be note that the immune responses of mice may not be extrapolated to other species. Therefore, further studies in sheep are required to evaluate the ability of the two proteins to control chronic non-progressive pneumonia.

## Supporting Information

S1 Fig Amplification of EF-Tu and HSP70 gene of *M*. *ovipneumoniae* by overlap extension PCR and construction of the pET-28a (+)-EF-Tu and pET-28a (+)-HSP70 plasmids. (A) Extension PCR of the first round. Overlap extension PCR was used to mutate the A of UGA into G in the gene sequence of EF-Tu and HSP70 genes from *M*. *ovipneumoniae* for normal expression of these two genes. Lane M, BM5000 DNA Marker (BioMed, Beijing, China); Lane 1, PCR amplification fragment MoEFTu-AB by using primer 1 and 2; Lane 2, PCR amplification fragment MoEFTu-CD by using primer 3 and 4; Lane 3, PCR amplification fragment MoHSP70-AB by using primer 5 and 6; Lane 4, PCR amplification fragment MoHSP70-CD by using primer 7 and 8; (B) PCR amplification of the second round; MoEFTu-AB, MoEFTu-CD and MoHSP70-AB, MoHSP70-CD were used as the amplification templates in the second round. Lane M: BM5000 DNA Marker (BioMed, Beijing, China); Lane 1. PCR amplification fragment mEF-Tu by using primer 1 and 3; Lane 2. PCR amplification fragment mHSP70 by using primer 5 and 8; (C) Identification of the recombinant expression plasmids by double digest of restriction enzyme. Full length fragments were cloned into the PET-28a (+) prokaryotic expression vector to construct the pET-28a (+)-EF-Tu and pET-28a (+)-HSP70 expression plasmids. Lane 1, pET-28a (+)-mEF-Tu plasmid digested by NcoI and XhoI; Lane 2, pET-28a (+)-mHSP70 plasmid digested by NcoI and XhoI.
(TIF)

S2 Fig Immunoreactivity analysis of *M*. *ovipneumoinae* EF-Tu and HSP70. Whole-cell proteins of *M*. *ovipneumoinae* strain Y98 (lane 1), *M*. *ovipneumoinae* wild strain Mo-1 (lane 2), *M*. *Arginini* (lane 3), *M*. *hyopneumoniae* (lane 4), *M*. *mycoides* subsp. *capri* (lane 5), *M*. *mycoides* subsp. *capri LC* (lane 6), *M*. *capricolum* subsp. *capricolum* (lane 7), *M*. *agalactiae* (lane 8), *M*. *bovis* (lane 9), *Brucella ovis* (lane 10) and *S*. *Dublin* (lane 11) were separated by 12% SDS-PAGE (A), blotted onto a PVDF membrane and subjected to the following Western blot analysis with mouse anti-rEF-Tu/anti-rHSP70 sera (B).
(TIF)
