# Correction: Elongation Factor Tu and Heat Shock Protein 70 Are Membrane-Associated Proteins from *Mycoplasma ovipneumoniae* Capable of Inducing Strong Immune Response in Mice

**DOI:** 10.1371/journal.pone.0189562

**Published:** 2017-12-07

**Authors:** Fei Jiang, Jinyan He, Nalu Navarro-Alvarez, Jian Xu, Xia Li, Peng Li, Wenxue Wu

There is an error in the third sentence of the Introduction. It references the following symptoms of *Mycoplasma ovipneumoniae*: “Progressive wasting, spasmodic cough, diarrhea and anemia are some of the characteristic symptoms of the disease.” The correct sentence is: “Progressive wasting and spasmodic cough are some of the characteristic symptoms of the disease.”
