# Supplementary figures and images for: Elongation Factor Tu and Heat Shock Protein 70 Are Membrane-Associated Proteins from Mycoplasma ovipneumoniae Capable of Inducing Strong Immune Response in Mice
Source: PLoS One. 2016 Aug 18;11(8):e0161170. doi: 10.1371/journal.pone.0161170 (PMC4990256; doi:10.1371/journal.pone.0161170)

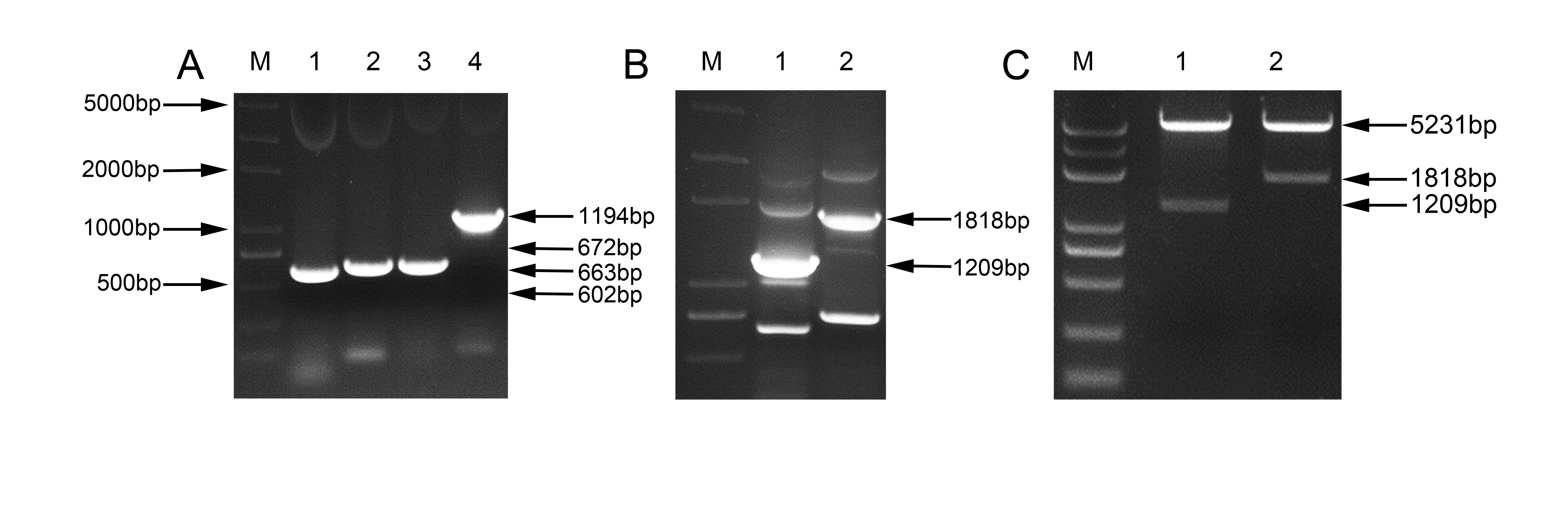

Supplement: S1 Fig — (A) Extension PCR of the first round. Overlap extension PCR was used to mutate the A of UGA into G in the gene sequence of EF-Tu and HSP70 genes from M. ovipneumoniae for normal expression of these two genes. Lane M, BM5000 DNA Marker (BioMed, Beijing, China); Lane 1, PCR amplification fragment MoEFTu-AB by using primer 1 and 2; Lane 2, PCR amplification fragment MoEFTu-CD by using primer 3 and 4; Lane 3, PCR amplification fragment MoHSP70-AB by using primer 5 and 6; Lane 4, PCR amplification fragment MoHSP70-CD by using primer 7 and 8; (B) PCR amplification of the second round; MoEFTu-AB, MoEFTu-CD and MoHSP70-AB, MoHSP70-CD were used as the amplification templates in the second round. Lane M: BM5000 DNA Marker (BioMed, Beijing, China); Lane 1. PCR amplification fragment mEF-Tu by using primer 1 and 3; Lane 2. PCR amplification fragment mHSP70 by using primer 5 and 8; (C) Identification of the recombinant expression plasmids by double digest of restriction enzyme. Full length fragments were cloned into the PET-28a (+) prokaryotic expression vector to construct the pET-28a (+)-EF-Tu and pET-28a (+)-HSP70 expression plasmids. Lane 1, pET-28a (+)-mEF-Tu plasmid digested by NcoI and XhoI; Lane 2, pET-28a (+)-mHSP70 plasmid digested by NcoI and XhoI. (TIF) [file pone.0161170.s001.tif]

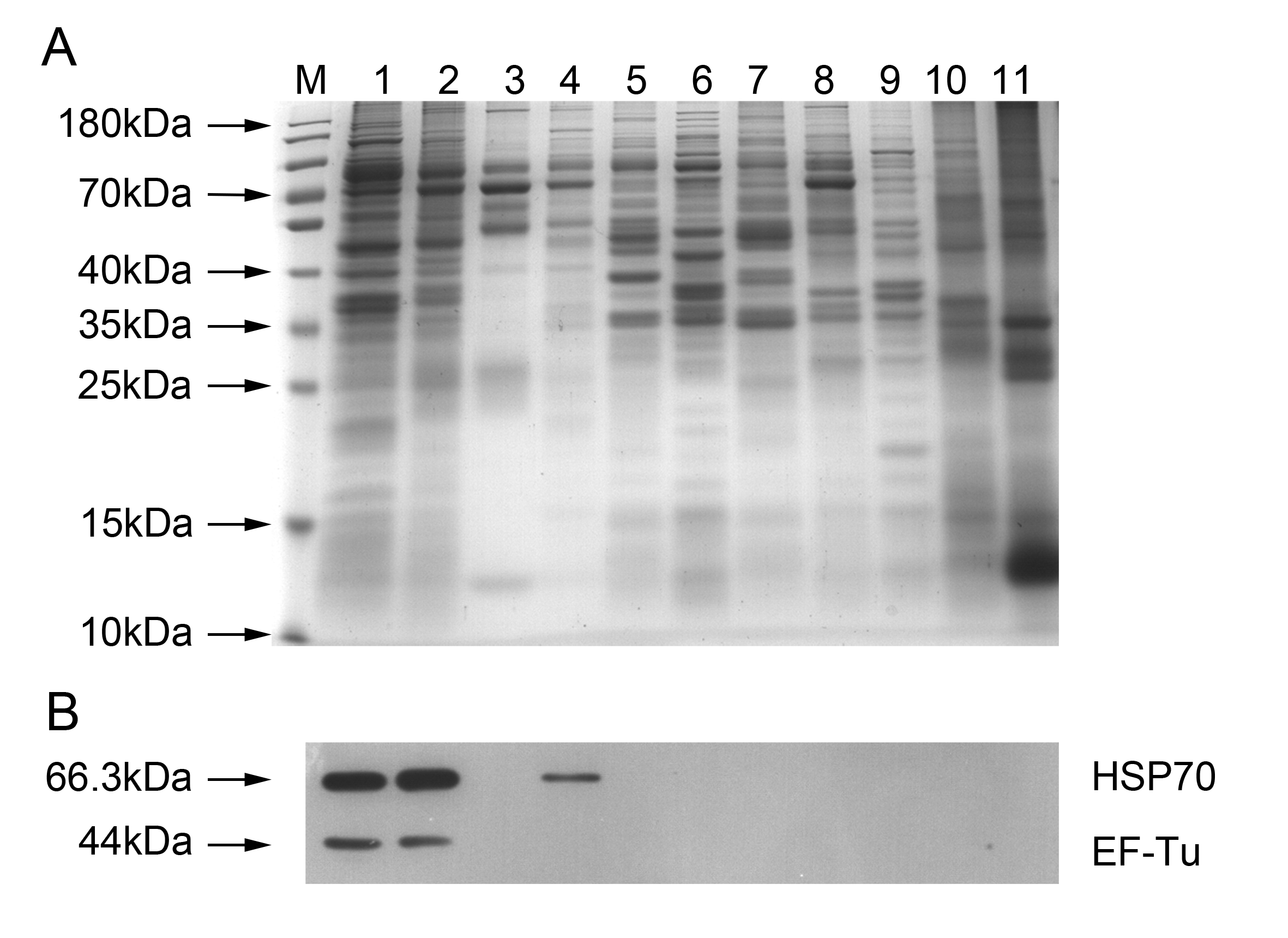

Supplement: S2 Fig — Whole-cell proteins of M. ovipneumoinae strain Y98 (lane 1), M. ovipneumoinae wild strain Mo-1 (lane 2), M. Arginini (lane 3), M. hyopneumoniae (lane 4), M. mycoides subsp. capri (lane 5), M. mycoides subsp. capri LC (lane 6), M. capricolum subsp. capricolum (lane 7), M. agalactiae (lane 8), M. bovis (lane 9), Brucella ovis (lane 10) and S. Dublin (lane 11) were separated by 12% SDS-PAGE (A), blotted onto a PVDF membrane and subjected to the following Western blot analysis with mouse anti-rEF-Tu/anti-rHSP70 sera (B). (TIF) [file pone.0161170.s002.tif]
